# Separation at birth due to safeguarding concerns: Using reproductive justice theory to re‐think the role of midwives

**DOI:** 10.1111/birt.12842

**Published:** 2024-06-05

**Authors:** Kaat De Backer, Hannah Rayment‐Jones, Elsa Montgomery, Abigail Easter

**Affiliations:** ^1^ Department of Women & Children's Health, School of Life Course and Population Sciences, Faculty of Life Sciences & Medicine King's College London, St. Thomas' Hospital London UK; ^2^ Division of Methodologies, Florence Nightingale Faculty of Nursing, Midwifery & Palliative Care King's College London London UK

**Keywords:** child protection, critical midwifery studies, maternity care, reproductive justice theory, safeguarding

## Abstract

Separation at birth due to safeguarding concerns is a deeply distressing and impactful event, with numbers rising across the world, and has devastating outcomes for birth mothers and their children. It is one of the most challenging aspects of contemporary midwifery practice in high‐income countries, although rarely discussed and reflected on during pre‐ and post‐registration midwifery training. Ethnic and racial disparities are prevalent both in child protection and maternity services and can be explained through an intersectional lens, accounting for biases based on race, gender, class, and societal beliefs around motherhood. With this paper, we aim to contribute to the growing body of critical midwifery studies and re‐think the role of midwives in this context. Building on principles of reproductive justice theory, Intersectionality, and Standpoint Midwifery, we argue that midwives play a unique role when supporting women who go through child protection processes and should pursue a shift from passive bystander to active upstander to improve care for this group of mothers.

## Graphical abstract

Separation between mother and baby shortly after birth due to safeguarding concerns is a deeply distressing and traumatising event, for all involved. We investigated the prevalence and incidence of infant separation and its impact on mothers and babies. Building on principles of Reproductive Justice Theory, Intersectionality and Standpoint Midwifery, we critically reviewed the role of midwives in this complex issue.

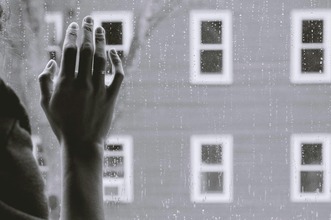

## 1 INTRODUCTION

**FIGURE 1:**
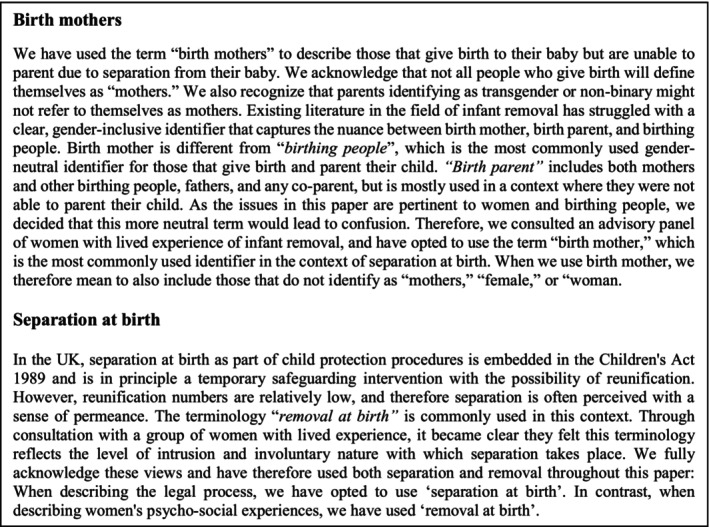
Notes on terminology.

In the United Kingdom, as in other countries around the world, a newborn baby can be removed/separated from its mother's care within a few days after the birth if there are serious concerns about its safety and well‐being Figure 1. National child protection legislation enables court cases to happen, sometimes within hours or days after a baby's arrival, when its mother is still recovering from birth. Child protection processes can be initiated during the pregnancy, often after a referral by midwives caring for the pregnant woman and her unborn baby. Separation at birth is seen as the highest sanction imposed by the State in families' personal lives^1^ and one of the most distressing aspects of contemporary midwifery practice.^2^ It is diametrically opposed to the women‐centered underpinnings of the midwifery profession and interrupts physiological processes that are beneficial to both mother and baby. However, midwives are often expected to align themselves to system‐driven procedures within social care and maternity services, impacting women‐centered care. This dual role of care provider for the woman and safeguarding the baby creates tension and leaves midwives in an uncomfortable and morally exposed position.^3^ Critical reflections and guidance around this issue are not routinely embedded in the midwifery curriculum, leaving midwives unprepared to navigate this moral maze. The support needs of these women can be complex and multi‐faceted, as many have experienced adversity and trauma. Yet, persistent stigmatizing attitudes in maternity services give little room for trauma‐informed approaches toward these mothers and can lead to unchallenged punitive processes with midwives as passive bystander.

## 2 INFANTS SEPARATED AT BIRTH: PREVALENCE AND INCIDENCE

**FIGURE 2:**
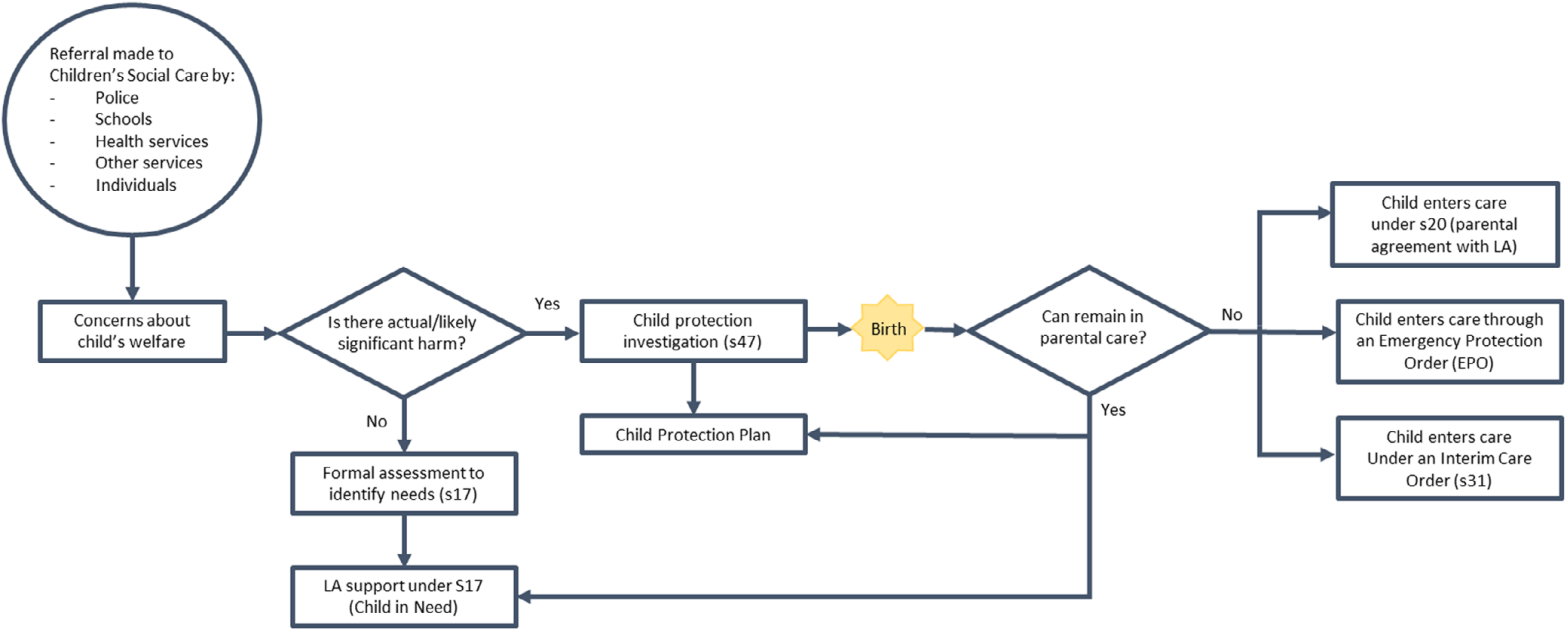
Social care involvement in the perinatal period. [Colour figure can be viewed at wileyonlinelibrary.com]

In the last decade in the United Kingdom, there has been an overall increase in social care involvement rates among children across all age groups.^4^ Social care involvement can vary depending on the level of risk to significant harm to a child and the level of urgency for local authorities to intervene. A full overview of social care involvement during the perinatal period, from referral to separation can be found in Figure 2.

The *Born into Care* research series^5^ investigated the incidence and prevalence of separation at birth, and defined “born into care” as those babies in State Care within the first week after birth.^5^ The average number of newborn babies in State Care through a court order increased in England from 2425 per year in 2012/2013 (or 34.9 babies per 10,000 live births) to 2914 in 2019/2020 (47.7 per 10,000 live births) and in Wales from 145 (41.1 babies per 10,000 live births) to 241 (68.3 per 10,000 live births) over the same period.^6^ Bilson and Bywaters^7^ have argued that the size of the issue has been underestimated as the Born into Care series only reported on children who were separated from their parents after a family court ruling. Their investigations show many more babies enter State Care by way of parental agreement with Children's Social Care, leading to the number of babies born into care being 44% higher than reported by the Born into Care research series. Similarly high rates of infants in child protection procedures and entering the foster care system can be found in other high‐income countries, such as Australia, New Zealand, and the United States, with Black, Brown, and Indigenous children disproportionately overrepresented in the child welfare system.^8^, ^9^, ^10^

The well‐being and safety of children are at the core of child protection processes. A meta‐analysis by Goemans et al.^11^ highlighted that the option of remaining at home with birth parents with additional support, and the option of foster care placement, both have their own risks and benefits. At risk children in both groups had poorer developmental outcomes than children in the general population (not at risk). However, when comparing children in foster placements with at risk children who remained at home, both faced comparable serious developmental challenges in cognitive, adaptive, and behavioral functioning.^11^ This makes decisions about removing a child from its parents contentious and challenging.

## 3 WOMEN FACING SEPARATION AT BIRTH: CHARACTERISTICS AND OUTCOMES

Evidence has shown that many women facing separation from their baby have experienced childhoods marked by neglect, abuse, and trauma. Over half of mothers were in State Care themselves at some point during their lives, demonstrating an intergenerational cycle of child removal and traumatic separation.^12^ Risk factors associated with having a child removed are varied^13^, ^14^ and presented in Table 1.

**TABLE 1:** Risk factors for parent‐infant separation due to safeguarding concerns.

Risk factors associated with having a child removed from parental care	
Low socioeconomic status Receipt of welfare benefits Single parenthood Ethnicity Age Disability Smoking in pregnancy Mental illness	Alcohol misuse Learning disabilities Parental exposure to adverse experiences during childhood Parental history of crime Acrimonious separation Patterns of multiple consecutive partners Intergenerational cycles of child maltreatment

The ongoing psychological sequelae of trauma and adversity have a profound and lasting impact. Recent studies found that women in care proceedings had higher rates of mental health needs prior to and during pregnancy than matched comparison groups.^15^, ^16^ Even before removal, they have higher rates of mental illness, including schizophrenia, depression, anxiety, substance use disorders, and suicide attempts.^17^ In addition to poorer mental health, birth parents face poorer physical health before the start of care proceedings, with outcomes worsening after child removal.^18^ Yet, despite these increased mental and physical health needs, antenatal care for this group of women often falls short, with higher rates of inadequate or no prenatal care for women who experienced removal of their baby compared to women with no previous history of child removal.^19^ Fear of their baby being taken into care can leave women reluctant to access antenatal services and share the full extent of their difficulties.^20^, ^21^, ^22^ Perceived judgement by healthcare professionals, including midwives, creates further barriers to accessing, engaging with, and receiving healthcare.^21^, ^22^, ^23^, ^24^ However, adequate support and timely intervention are proven to enable women who have experienced high levels of adversity and mental health difficulties to safely care for their children.^25^

The legacy of colonialism, slavery, and structural racism cannot be underestimated in this context and has been linked to current inequalities of racialized and Indigenous peoples in settler colonial countries such as Canada, Australia, and New Zealand.^26^ The continued denial of Indigenous people's sovereignty, and by extension their parental rights, has led to pervasive Indigenous health inequalities^27^, ^28^ and intergenerational, historical, and complex trauma in Indigenous families.^29^ Overall, ethnic inequalities in child protection, including in the United Kingdom, require further rigorous examination, taking into account underlying social circumstances and the complexity of the available epidemiological data.^30^

Removal at birth is a deeply distressing, intrusive, and emotionally impactful event, leaving women to feel ashamed, rejected, and stigmatized.^31^, ^32^, ^33^ Child removal has also been found to have a profound impact on maternal mortality and morbidity post‐removal.^17^ Women who have a child removed from their care have threefold increased mortality rates, both by avoidable causes (such as suicide)^34^, ^35^, ^36^ as well as by unavoidable causes (such as ischemic heart disease).^34^, ^35^ In the United Kingdom, the Confidential Inquiry into Maternal Deaths (MBRRACE‐UK) has highlighted that women with multiple adversities are particularly vulnerable and at increased risk of death by suicide or overdose in the year after birth.^37^, ^38^ Even more alarming, all teenagers who died by suicide in the United Kingdom in 2020 during pregnancy or the year after giving birth had their baby taken into care prior to their suicide.^38^

In the immediate postnatal period, women whose baby is taken into care face an acute psychosocial crisis, which can lead to a return to harmful coping strategies, such as misuse of drugs and alcohol.^33^, ^39^ Mothers who have their baby removed after birth have higher rates of postnatal depression compared to women with lower levels of social services involvement.^17^ One in four women will return to the courts within 10 years, often for new care proceedings after the birth of another baby, with the highest risk within the first 3 years of the initial proceedings.^40^ Young mothers, particularly those who have their first child as teenagers, are at a significantly high risk of returning to court.^41^ This sequence of rapid repeat pregnancies compounds previous trauma and loss^40^ and carries significant health risks for both mother and babies, such as preterm birth and low birth weight.^42^, ^43^ Separation from their mother at birth disrupts critical attachment processes essential to the formative stage of babies' brain development, impacting the long‐term outcomes of these children.^44^

## 4 REPRODUCTIVE JUSTICE THEORY

Rooted in the SisterSong Black feminist movement in the United States in the 90s, reproductive justice theory is a contemporary feminist theoretical framework that brings *reproductive rights* together with *social justice* to achieve *reproductive justice*. It upholds three primary principles that are considered to be human rights for all: (1) the right *not* to have a child; (2) the right to *have* a child; and (3) the right to *parent* children in safe and healthy environments. In addition, reproductive justice theory demands sexual and bodily autonomy and gender freedom to every human being.^45^ Reproductive justice theory bridges the gap between traditional feminist theories (problematizing gender) and critical race theories (problematizing race). Its central belief is that systemic inequality and power dynamics shape people's options and decision‐making around childbearing and parenting, and thus affect any groups of vulnerable women in particular.^46^ In 2006, a collective of reproductive justice scholars defined how reproductive justice theory can be applied outside its original context of the Black African feminist movement in the United States and invited scholars or activists to embrace, adapt, and re‐invent the reproductive justice framework.^47^ The framework is particularly relevant to examine societal prejudices that impact pre‐referral decision‐making and systemic failings of child protection services, the criminal justice system, and healthcare services. Race, class, and gender biases influence decisions in these systems and in the spaces where these systems meet.^45^ The right to have a child is intertwined with the right to parent children in safe and healthy environments, and one cannot be achieved without the other. As long as women (and families) are enduring poverty, racism, misogyny, classism, among many other forms of oppression, and are penalized for it through decisions about their parental capacities, these rights can never be fulfilled. We argue, that denying women the right to parent by removing their infant, while failing to acknowledge and address their precarious living conditions, limited access to support services, and systemic oppression, is undermining women's reproductive rights on both accounts.

## 5 INTERSECTIONALITY

Racial and ethnic disparities, both prevalent in maternity care and child protection referrals, can be understood through a lens of intersectionality, a concept stemming from the Black feminist movement and first coined by Kimberlé Crenshaw in 1989. Intersectionality describes the *confluence* of oppressions, based on the multiple identities one holds in society.^48^ Initially, Crenshaw focused on the oppressions based on race (i.e., racism) or gender (i.e., sexism), to explain the unique experience of women of color and their experiences of oppression and discrimination. Recent studies applying an intersectional lens most commonly incorporate identities of race, ethnicity, sex, gender, class, and ability. An intersectional approach can explain not just health inequalities in the context of maternity care,^49^ but also disparities in social care intervention.^50^, ^51^

When looking at health inequalities through a lens of racist and colonial oppression, in many settler colonial countries, women of racialized and Indigenous backgrounds are disproportionately subjected to scrutiny and surveillance, such as toxicology screening during pregnancy and at birth, explaining an overrepresentation in referral rates to child protection agencies.^52^ Historically, generations of Black and Indigenous communities had their children forcibly removed, either through structures linked to slavery, or by governments, churches, and welfare bodies, leading to so‐called “Stolen Generations”.^53^, ^54^ To this day, perpetuating racism toward these communities still accounts for disproportionate removal rates of ethnic minority and/or Indigenous babies.^26^, ^55^ In contrast, in the United Kingdom, the rapid increase of babies born into care has been mostly linked to a decade of austerity policies, marked by a general climate of economic hardship, shrinking budgets for children's social care, and funding cuts to prevention and support services for struggling families, including closure of specialist domestic violence and drugs and alcohol services.^50^, ^56^, ^57^ As such, regions with higher levels of deprivation have been found to have both larger proportions of babies born into care, and higher rates of growth.^6^, ^7^, ^58^ While poverty has been strongly associated both with child abuse and neglect as well as with intervention rates,^57^ it is not sufficient to explain the existing racial, ethnic, and social disparities in referrals to UK child protection services. Evidence has exposed a complex picture, requiring an intersectional lens: when comparing referral rates at different levels of deprivation in the United Kingdom, some ethnic minority groups are found to be underrepresented in poorer neighborhoods and overrepresented in affluent neighborhoods.^50^ This could suggest Black or Brown families are disproportionately scrutinized and referred in affluent areas compared to their White counterparts, a trend also seen in other high‐income countries.^8^, ^9^, ^10^ Racial and ethnic disparities have been widely evidenced in maternity systems across the world, with higher mortality and morbidity rates among Black and Brown women^38^, ^53^, ^54^, ^59^ and systematic racism remaining a pressing issue in maternity care.^26^, ^55^

Societal beliefs around motherhood in high‐income countries idealize the role of the traditional hetero‐normative, white, middle‐class woman, who adheres to prescribed norms of maternal sacrifice, both in terms of caring for her children as well as providing for her husband.^60^, ^61^ In par with this ideal, women who do not meet these standards, for example, women of poor economic class, ethnic minorities, or single mothers, are deemed less “suitable” or “fit” to be a mother, and are subjected to scrutiny.^33^, ^61^ This is illustrated by the practices in Irish mother and baby homes during the 20th century, where unmarried and/or destitute women and girls were forcibly separated from their babies, who often were adopted against their mother's wishes. Such eugenic beliefs toward motherhood, deeply rooted in colonialism and pauperism, continue to go unchallenged, not just in society, but by extension in the health and social care workforce. They seep into clinical care and into decisions about who to refer to child protection services and who not to. The midwifery workforce, with its dual role as care provider to women and referrer of the (unborn) baby to children's social services, has to undertake critical reflections on its conscious and unconscious biases, and on the role midwives play in these unacceptable disparities.

## 6 RE‐THINKING THE ROLE OF MIDWIVES–STANDPOINT MIDWIFERY PRINCIPLES AND RECOMMENDATIONS

Ashley et al.'s^62^ invitation to critically reflect on the role of midwifery in order to dismantle institutionalized racism and injustice in sexual, reproductive, maternal, and newborn healthcare is timely and essential. The authors' approach to Midwifery as a Standpoint Epistemological position acknowledges midwives as “situated” or “advantaged knowers,” whose standpoint can reveal social (ir)regularities and arrangements unbeknown to others.^63^, ^64^

Midwives have a unique role and can be agents for change in child protection processes. A first, crucial step is to acknowledge how systemic racism and colonialism in maternity care perpetuates maternal health disparities and discrepancies between social care referrals and removals of babies from Indigenous and racialized groups. This recommendation builds on earlier calls to the worldwide healthcare community to come to terms with decolonialization as a step toward the transformation of Indigenous and racialized health inequalities.^27^ The historical evolution of the midwifery profession in this context is turbulent. Traditional and community‐based (lay) midwives have been century‐long advocates for Indigenous and Black women's reproductive rights.^65^, ^66^ However, with the medicalization of childbirth, labor and birth care became dominated by the white male gaze, and lay midwives and birth attendants were marginalized.^65^ To this day, in some US states midwives continue to risk prosecution for providing state‐outlawed midwifery care.^65^, ^67^ Midwives from racialized and Indigenous groups have become a minority in the midwifery workforce, which is further contributing to disparities in maternal health outcomes of mothers from these communities.^26^ A workforce‐wide reckoning with the legacy of colonialism, slavery, and racism is therefore crucial to advocate for the reproductive rights of women from racialized and Indigenous groups. Important steps to decolonize midwifery and the midwifery curriculum are being made, for instance by the “Decolonizing Midwifery Toolkit” from the Royal College of Midwives (UK).^68^ However, such efforts need to be accelerated and extended to both the existing midwifery workforce, their interdisciplinary healthcare colleagues and student midwives.

Second, midwifery continuity of care has been shown to improve both bio‐medical and psycho‐social outcomes of women and babies,^69^ especially for women facing multiple adversities.^70^ Even when reporting to child protection agencies is required, the relational aspect of midwifery continuity of care makes it more likely to withstand a temporary rupture, and to facilitate repair and continued engagement. In addition, forming multi‐disciplinary relationships based on trust and respect with professionals in child protection services strengthens the role of midwives from bystander to upstander, from *reporting on* women to *advocating for* women in their care.

Third, trauma‐informed maternity care has found its way to the public health priorities agenda,^71^ yet often remains unapplied in clinical practice. For maternity care to be truly trauma‐informed, four assumptions have to be upheld: (1) Staff should acknowledge the impact of previous or current trauma on people's maternity experiences and their opportunities in life, including on developing healthy coping strategies and loving, sustainable relationships; (2) staff are able to recognize the signs of trauma, irrespective of disclosure; (3) maternity services have organizational policies and procedures in place that consider experiences of trauma, both among service users and its workforce; and (4) services take active steps to avoid re‐traumatization of service users and staff members at all times.^71^, ^72^ When these principles are upheld, maternity services and their staff can become active upstanders by challenging current biases and dismantling punitive, penalizing, and degrading attitudes toward these mothers. By doing so, women presenting with substance dependency, abusive relationships, previous removals of children, etc., will no longer be viewed as “failed mothers(−to‐be)” who made deliberate decisions to retreat to drugs as a coping strategy, but as survivors of trauma and adversity, requiring compassionate care and support instead of judgment and retribution. By adopting a trauma‐informed approach when caring for women facing removal of their infant, midwives can realize the healing and empowering potential of midwifery care. However, such system change requires a broader policy overhaul, which has already started to take shape in the United Kingdom by policy frameworks such as Core20PLUS5^73^ and the NHSE Equity and Equality Hub.^74^ This, in combination with guidelines such as the Born into Care Best Practice guidelines for when the state intervenes at birth,^75^ Birth Companion's Birth Charter for women with involvement from children's social care^76^ and partnerships with key stakeholders, such as the NHS Race and Health Observatory^77^ can further support the policy drive and professionals' behavior change to advance equity.

## 7 CONCLUSION

In this paper, we applied an intersectional lens to account for biases based on race, gender, class, and societal beliefs around motherhood and disparities in health outcomes and child protection practices. Building on the principles of reproductive justice theory, we argue that removing a child from its mother, while leaving the reason that gave cause to this decision unaddressed, breaches both the right to parent a child as well as the right to parent children in safe and healthy circumstances. The current clinical reality reduces the role of midwives to that of a reporting agent and passive bystander, therefore potentially contributing to the re‐traumatization of women facing multiple adversity and trauma. Instead, we argue for a critical re‐thinking of the role of midwives when caring for women facing removal of their baby. In line with the principles of Standpoint Midwifery, a shift from passive bystander to active upstander should be pursued, through acknowledging the role of colonialism and racism in maternity care, offering community‐based midwifery continuity of care, and embedding trauma‐informed approaches in maternity care systems.

## FUNDING INFORMATION

Kaat De Backer (NIHR Doctoral Research Fellow, NIHR302565) is funded by the National Institute for Health and Care Research for this research project, as well as Dr Hannah Rayment‐Jones (NIHR303183). Dr Abigail Easter (King's College London) is currently supported by the National Institute for Health and Care Research Applied Research Collaboration South London [NIHR ARC South London] at King's College Hospital NHS Foundation Trust. The views expressed in this publication are those of the authors and not necessarily those of the NIHR, NHS, or the Department of Health and Social Care.

## CONFLICT OF INTEREST STATEMENT

Professor Jane Sandall, who kindly provided feedback on the manuscript, is a member of the editorial board of Birth.

## Data Availability

Data sharing is not applicable to this article as no new data were created or analyzed in this study.
